# Soluble ST2 in coronary artery disease: Clinical biomarkers and treatment guidance

**DOI:** 10.3389/fcvm.2022.924461

**Published:** 2022-09-26

**Authors:** Junyan Zhang, Zhongxiu Chen, Min Ma, Yong He

**Affiliations:** Department of Cardiology, West China Hospital of Sichuan University, Chengdu, China

**Keywords:** sST2, coronary artery disease, myocardial infarction, LVR, management

## Abstract

The IL-33/ST2 L signaling pathway is involved in the pathophysiological processes of several diseases and mainly exerts anti-inflammatory and antifibrotic effects. Soluble suppression of tumorigenicity 2 (sST2), which serves as a competitive inhibitory molecule of this pathway, is a member of the interleukin (IL)-1 family, a decoy receptor for IL33, thought to play a role in cardiac remodeling and the inflammatory process. However, the association between sST2 and coronary artery disease (CAD), one of the most common causes of heart failure, is still being explored. We therefore reviewed the research on sST2 in the field of CAD, including reflecting the atherosclerosis burden, predicting no-reflow, predicting prognosis, responding to myocardial remodeling, and guiding management, hoping to provide cardiologists with new perspectives.

## Introduction

**Figure 1:**
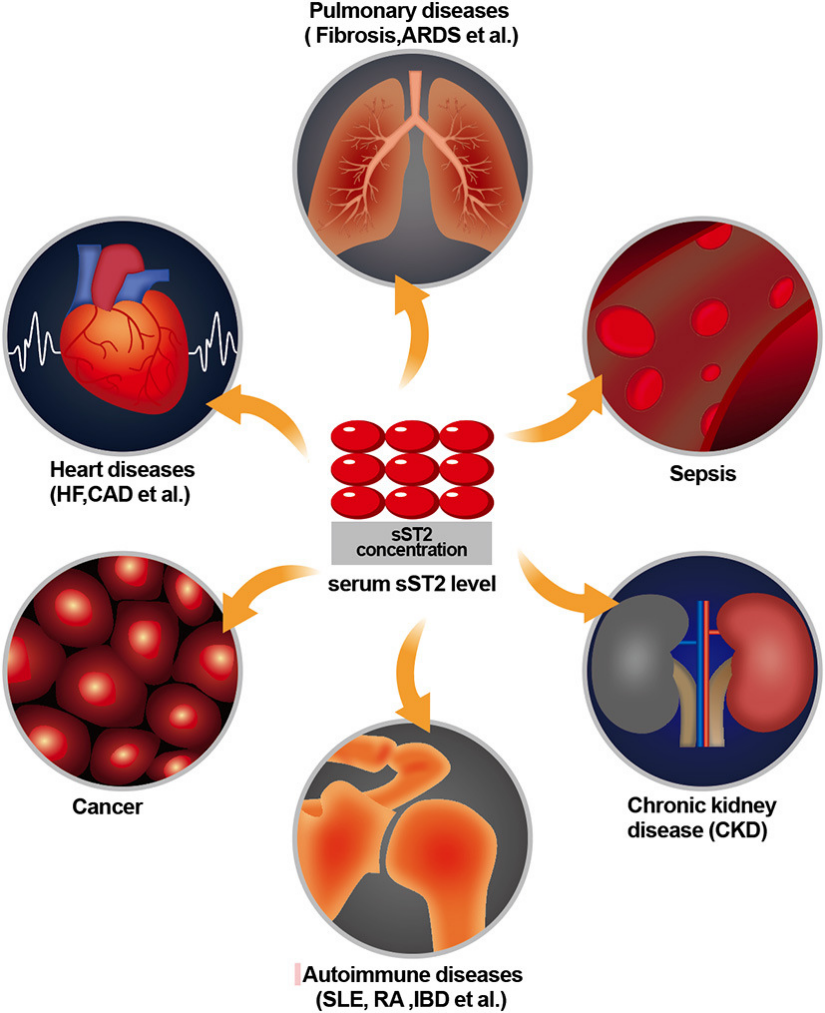
Role of sST2 in diseases of various systems.

Suppression of tumorigenicity 2 (ST2) is a member of the interleukin 1 (IL-1) receptor family and is formally known as interleukin 1 receptor-like 1 (IL1RL-1). It was first described in 1989 but remained an orphan receptor mainly related to immune and inflammatory diseases for years (1). In 2005, ST2 was reported to be expressed in cardiac cells in response to myocardial stress, and interleukin 33 (IL-33) was reported to be the ligand of ST2 (2). Since then, its role in cardiovascular diseases has been of great concern. The ST2 gene is located on human chromosome 2q12 and encodes two main protein isoforms: transmembrane receptor (ST2 L) and truncated soluble receptor (sST2). The interaction between IL-33 and ST2 L mediates anti-inflammatory and antifibrotic effects (3). For instance, the activation of mitogen-activated protein kinase (MAPK) and nuclear factor (NF-kB) signaling originates from the binding of IL-33 to ST2 L, which produces various downstream effects in target cells in the presence of additional interleukin-1 receptor accessory protein (IL-1RacP) receptor protein molecules. In contrast, when IL-33 binds to sST2, it prevents and blocks these effects (4). While sST2 can be secreted into the circulation and functions as a decoy receptor for IL-33, it is unavailable to ST2 L, which abolishes the cardioprotective effects of IL-33/ST2. Meanwhile, ST2 is established as a selective marker of T helper type 2 (Th2) lymphocytes, which are also expressed on mast cells, epithelial cells, endothelial cells, smooth muscle cells, neonatal cardiac fibroblasts, and cardiac myocytes. More specifically, sST2 can serve as a non-invasive diagnostic and prognostic marker for lung, gastric, breast, pancreatic, colon, and other cancers (5–7); can be present in diseases associated with a predominantly Th2 response, such as asthma, pulmonary fibrosis, and rheumatoid arthritis (8, 9); can be useful for risk stratification and prediction of prognosis in patients with suspected sepsis (10, 11); and can enhance the development of fibrosis, hypertrophy, remodeling of the heart muscle, and progression of heart failure (12, 13) (Figure 1). Although sST2 is involved in the pathophysiology of several diseases, an increasing number of recent studies have focused on heart disease, especially heart failure. Since sST2 is less influenced by age and renal insufficiency than N-terminal pro-B-type natriuretic peptide (NT-proBNP) and high-sensitivity troponin T (hs-TnT) (14, 15), it has been entered into guidelines in heart failure and considered to provide additive prognostic value and as a guide to therapy decision-making of heart failure (16).

**Figure 2:**
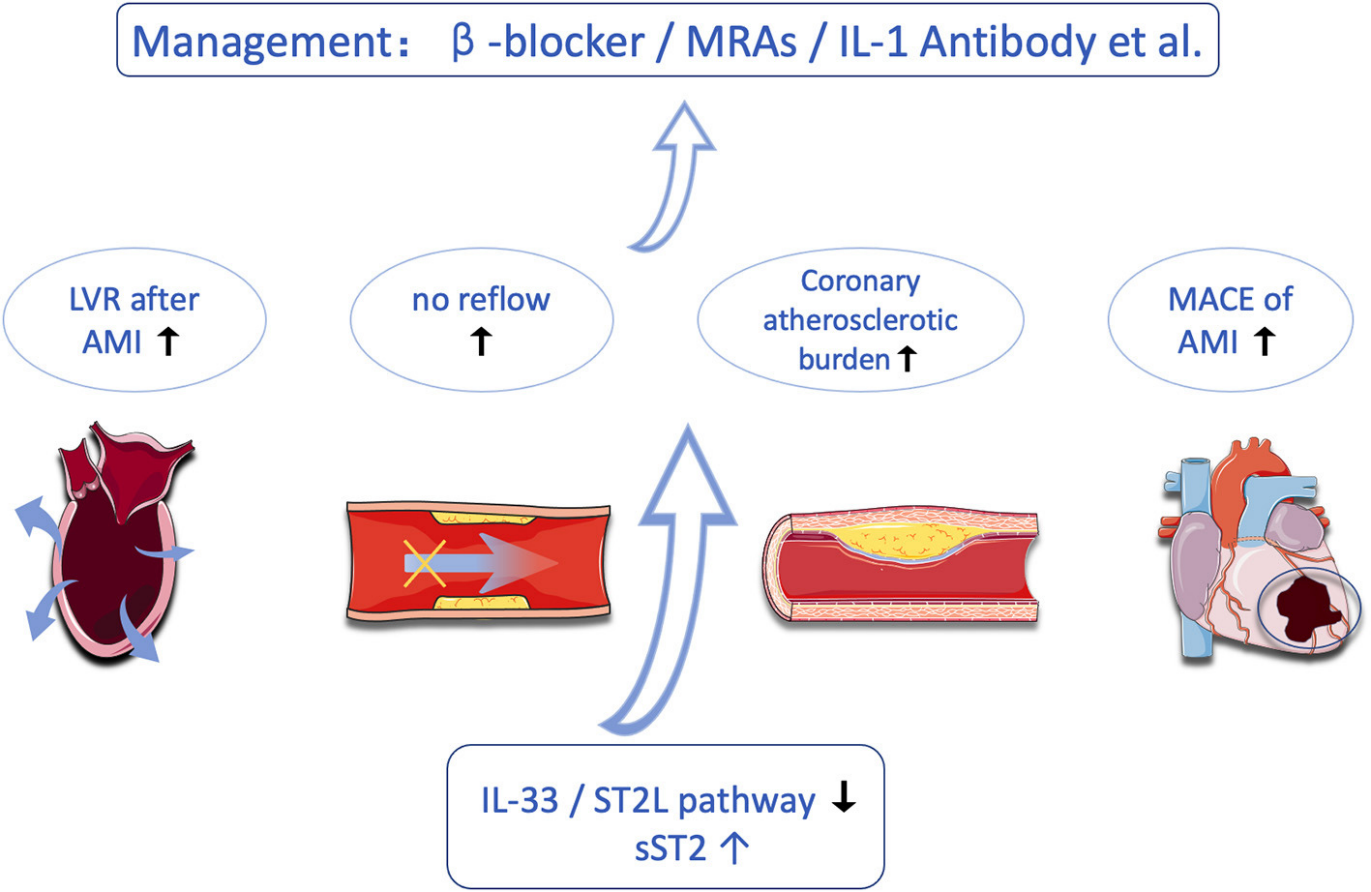
Research progress on sST2 in the field of coronary heart disease.

Coronary artery disease (CAD), especially acute coronary syndrome (ACS), is one of the most common causes of mortality throughout the world, despite technological improvements, new drugs, and an increasing level of awareness (17, 18). In fact, timely diagnosis allows physicians to stratify their patients by risk and consequently provide them with the opportunity to select appropriate treatments. Biomarkers that help refine diagnosis, risk stratification, and prognostic assessment are needed. In recent years, the role of ST2 in the pathophysiology of CAD and the clinical value of this biomarker in acute ST-segment elevation myocardial infarction (STEMI) have broadly expanded. In this study, we aimed to reappraise the current knowledge on sST2 in CAD (Figure 2).

## Association with coronary atherosclerotic burden

The “inflammatory hypothesis” of atherosclerosis postulates that inflammatory cell signaling drives the formation, growth, and ultimately the instability of atherosclerotic plaques, setting up the substrate for the thrombotic response that causes myocardial damage or infarction (19). In the arterial wall, the interaction between IL-33 and ST2 L directs the immune response toward a T helper 2, macrophage 2 phenotype, limiting plaque inflammation and evolution. In contrast, sST2 blocks the protective effects of IL-33 on atherosclerotic plaques by sequestering IL-33 (20).

Pfetsh et al. observed a strong correlation between elevated sST2 levels and inflammatory markers (hs-CRP and IL-6) that may reflect the presence of chronic inflammation in the pathophysiology of atherosclerosis (21). In addition to being closely related to the progression of atherosclerotic lesions, sST2 has been reported as a biomarker for the stability and complexity of coronary atherosclerosis. Previous studies have shown that an increased sST2 level is a strong marker of increased risk for mortality and adverse cardiac events, such as recurrent MI and stroke, in patients with AMI (22). Zhang et al. revealed that plasma sST2 levels were significantly higher in ACS patients with complex lesions than in those with simple lesions, which indicated that sST2 may be a new marker for assessing the stability and complexity of atherosclerotic plaques (23). However, the above study also showed that there were no correlations between plasma sST2 level and stenosis severity, measured by the number of culprit vessels and Gensini score. Dieplinger et al. came to the same conclusion as above, which elucidated that sST2 was not related to the angiographic severity of CAD (24). The reason for higher sST2 levels in patients with complex lesions than in patients with simple lesions in ACS was explained in the study of Demyanet et al., which showed that sST2 may play a role in the development of vulnerability and plaque rupture, which occurs most predominantly in the ACS population (25).

Based on the above studies, Luo et al. prospectively enrolled 120 patients to assess plaque vulnerability by coronary computed tomography angiography (CCTA) (26). Their study showed that higher serum sST2 levels were associated with higher plaque vulnerability. However, the two prevailing intracoronary imaging techniques in clinical practice, IVUS, which allows macroscopic visualization of the structure of the entire vessel wall, and OCT, which allows microscopic visualization of the subtle structure of the wall, complement each other and can be used as an important tool for identifying vulnerable plaques (27–29). Therefore, studies using IVUS and OCT as outcomes should be conducted to further validate the correlation between sST2 and plaque vulnerability.

Meanwhile, the coronary artery calcium score (CACS) is a marker of atherosclerotic plaque burden and an independent predictor of coronary events. In the study by Oh et al. (30), researchers enrolled 456 subjects to illustrate that, compared with hsCRP, sST2 does not improve net reclassification for predicting a high-risk CACS, defined as CACS ≥300 Agatston units. Overall, sST2 may reflect the atherosclerotic burden in unstable, complex atherosclerotic lesions, but further studies are needed to focus on this issue.

## Predict no-reflow phenomenon after percutaneous coronary intervention

The no-reflow phenomenon is defined as insufficiency of myocardial perfusion despite the mechanically responsible lesion being opened. The no-reflow phenomenon rate can reach as high as 50% in ACS patients (31) and restrain the positive effects of percutaneous coronary intervention (PCI). As there is limited treatment of no-reflow, it is more important to prevent it from occurring (32). Clinicians are committed to finding markers or clinical conditions that can predict no-reflow. It was demonstrated that sST2, a biomarker related to inflammatory activity, is one of the independent predictors of the no-reflow phenomenon in STEMI patients undergoing primary PCI. Somuncu et al. (33) included 379 patients who underwent PCI treatment for STEMI to determine the relationship between sST2 and the no-reflow phenomenon. Higher levels of sST2 patients had a significantly higher level of no-reflow compared with lower levels of sST2 (OR: 2.741 CI 95% 1.433–5.244, *p* = 0.002). Furthermore, after adjustment for potential confounders, it was found that being in a high-sST2 group was one of the independent predictors of no-reflow [area under the curve (AUC), 0.699; 95% confidence interval (CI), 0.65–0.75; *P* < 0.001].

Since the above study showed that sST2 can predict the no-reflow phenomenon in STEMI patients, experts then turned their attention to non-ST-segment elevation acute coronary syndrome (NSTE-ACS). Zhang et al. (34) revealed similar results, which pointed out that although the predictive ability was low, sST2 had a predictive value for no-reflow [area under the curve (AUC), 0.662; 95% confidence interval (CI), 0.53–0.79; *P* = 0.015]. It also had independent predictive value after adjusting for confounding factors [odds ratio (OR), 3.802; 95% CI, 1.03–14.11; *P* = 0.046]. However, both studies were single-center studies, and the number of patients included was relatively small. More importantly, they only detected the sST2 level of patients at the time of admission but did not observe subsequent changes. Thus, more high-quality trials should be carried out to determine the relationship between sST2 and no-reflow.

## As a prognostic biomarker of CAD

Shimpo et al. demonstrated that sST2 levels can predict mortality and heart failure in MI patients by extracting sST2 from the serum of 810 AMI patients (35). Then, Sabatine et al. measured ST2 at baseline in 1,239 patients with STEMI from the CLARITY-TIMI 28 trial, which showed that a high baseline ST2 level, irrespective of baseline features and NT-proBNP, is a strong predictor of cardiovascular mortality and heart failure in STEMI, and the combination of ST2 and NT-proBNP greatly enhances risk stratification (36). At the same time, studies have shown that sST2 is also an independent predictor of future death or heart failure in patients with acute chest pain in the emergency department (37). In contrast, sST levels did not predict the occurrence of MACEs in STEMI patients in the study by Kim et al. (38). The reason for this phenomenon may be that sST2 levels begin to rise at 3 h after STEMI and peak at 12 h. The length of time after AMI onset to reperfusion affects myocardial injury, which is associated with an increase in biomechanical strain, leading to higher sST2 levels. The median time from onset to PCI for patients in this study was 2.7–2.8 h, which is shorter than in other studies (38). These patients, on the one hand, had a short period of myocardial ischemia and may not have been severely injured; on the other hand, they may have had an earlier measurement of sST2, resulting in sST2 not yet being elevated to the desired level. As in STEMI patients, the prognostic predictive role of sST2 in patients with chronic coronary artery disease (SCAD) remains controversial. The 13-year follow-up results of the KAROLA study suggest that sST2 levels can be an independent predictor of mortality in SCAD patients but do not predict non-fatal cardiovascular events (21). Similarly, the results from the Ludwigshafen Risk and Cardiovascular Health Study also elucidated that increased sST2 levels were an independent predictor of long-term all-cause mortality in patients with SCAD (24). However, Demyanets et al. came to the opposite conclusion, stating that sST2 was not associated with mortality in SCAD patients, despite a strong relationship with mortality in STEMI patients (25). Hughes et al. also showed that sST2 does not function as a predictor of cardiovascular events in the general population (39). The reason for this discrepancy may be because the latter study included not only patients with unstable coronary plaque but also patients with stable coronary artery disease, which interfered with the results.

Although many studies have confirmed that sST2 has a strong predictive effect on the prognosis of heart failure, the number of studies on sST2 and the prognosis of CAD, both in ACS and chronic coronary syndrome (CCS), is limited, and their findings are controversial. Therefore, more studies should be conducted in the future to further explore the prognostic effect of sST2 on CAD.

## Reflect left ventricular remodeling after acute myocardial infarction

Left ventricular remodeling (LVR) refers to changes in the shape and size of the whole left ventricle after acute myocardial infarction (AMI). Important pathological features of postinfarction LV remodeling include infarct expansion, myocardial hypertrophy, cardiac fibrosis, and ventricular dilation, which are mainly due to inflammatory responses and neuroendocrine activation (40, 41). The development of adverse LVR after AMI remains a significant problem despite current achievements in invasive and pharmacological treatment (40).

Meanwhile, ST2 regulates the expression of proinflammatory cytokines from macrophages and prevents uncontrolled inflammatory reactions in the MI region. The sST2 level could be responsible for myocardial fibrosis and LVR, which could affect the prognosis after MI (40, 42). By constructing a mouse model of MI, Ghali et al. illustrated that IL-33 administration was associated with deterioration of cardiac function and ventricular remodeling. This study validated the role of the IL-33/ST2 axis in LVR after MI and laid the theoretical foundation for subsequent clinical studies (42). Thus, several studies using cardiac magnetic resonance (CMR) or echocardiography (ECHO) as a measure of LVR have been performed to demonstrate the relationship between sST2 and LVR after MI. Weir et al. included 100 patients with AMI for whom serum biomarkers were measured and CMR scans were performed and demonstrated a direct relationship between sST2 and LVR (43). It was also shown that sST2 was higher in individuals with microvascular obstruction (MVO), which is related to a more significant LVR and a poorer cardiovascular prognosis following AMI (44, 45). Kercheva et al. reached a similar conclusion from ECHO endpoints that the rise in serum sST2 was strongly associated with LVR after 6 months (46). Another similar study also found that sST2 levels correlated with both early LVR (<3 months) and late LVR (>3 months) (47). Based on these studies, using novel drugs that may antagonize this pathway, e.g., novel interleukin-1 monoclonal antibodies to antagonize the process of LVR, becomes theoretically possible, but this builds on more experiments demonstrating the causal relationship between sST2 and LVR (47).

Currently, mainly instrumental markers, such as the parameters of ECHO and CMR, are used to indicate the development of adverse LVR (48). The quest for a practical and reliable biomarker of adverse LVR, which would allow us to predict this disease in its early stages based on an exact assessment date, appears to be promising (49). Since both hemodynamic stress and an inflammatory nature are involved in the pathophysiological process of LVR, indicators that might reflect this process, such as NT-proBNP and hsCRP, have been of interest to cardiologists (36). Unlike NT-ProBNP, which responds to cardiac mechanical stress, sST2 reflects the degree of necrosis and inflammatory response of cardiomyocytes (50). Meanwhile, the dynamics of serum levels are different, which affects the timing and purpose of their clinical application. The level of sST2 decreased rapidly during the 7 days after MI; however, the level of NT-proBNP decreased effectively after the first 7 days (46). Then, Pecherina et al. performed a correlation analysis of echocardiographic parameters and serum biomarkers in patients with STEMI and preserved left ventricular ejection fraction, which showed that sST2 predicted LVR better than NT-proBNP (AUC 0.8 and 0.7, respectively), and several other biomarkers, such as matrix metalloproteinases (MMPs) and galectin-3, were also included in the study, illustrating that biomarkers combined with imaging findings can better predict the occurrence of LVR (51).

Moreover, several studies have found a link between sST2 and circulating aldosterone, implying that the IL-33/sST2 signaling system and the RAAS are linked (43). This raises the possibility of a direct role for the IL-33/sST2 system in the pathogenesis of postinfarction remodeling. Studies are already underway in this area, and the effects of drugs such as eplerenone, spironolactone, and beta-blockers on the IL-33/sST2 axis are gradually being discovered by cardiologists (52, 53), but more research is still needed in this area to find an optimal strategy to detect and cope with ventricular remodeling in an early stage after MI.

## As help for management of myocardial infarction

The IL-33/ST2 signaling pathway is involved in various adverse pathophysiological processes after infarction, such as fibrosis, inflammation, and hypertrophy, which are important targets for a variety of neuroendocrine antagonists currently available to improve the prognosis of MI patients. Xia et al. demonstrated that beta-blockers could inhibit fibrosis, reduce infarct size, and improve cardiac function by enhancing the IL-33/ST2 signaling pathway through the construction of an AMI mouse model and that this effect results in a decrease in serum sST2 levels (54). It is possible that measurement of serum sST2 levels may reflect the efficacy of beta-blockers in patients with AMI. Next, Gaggin et al. performed a *post-hoc* analysis of the PROTECT study in which the group of patients who received high-dose beta-blockers with low sST2 levels had the best prognosis, suggesting that sST2 levels can assist cardiologists in selecting the best treatment option for patients with MI (53). Like beta-blockers, mineralocorticoid receptor antagonists (MRAs) are also widely used as neuroendocrine antagonists that can inhibit myocardial remodeling and improve prognosis in patients with MI. Other studies have also shown that both eplerenone and spironolactone antagonize aldosterone and are able to enhance the IL-33/ST2 signaling pathway while decreasing serum sST2 levels. Therefore, the detection of serum sST2 levels can also reflect the efficacy of these drugs (52, 55).

## Conclusion and clinical perspectives

Soluble ST2 is a promising biomarker in cardiology, not only in heart failure but also in CAD. As a biomarker related to inflammation and fibrosis, sST2 has important clinical value in CAD, which may guide prognosis prediction, treatment plan selection, risk assessment, and long-term management of MI patients. In this article, we reviewed the use of sST2 in coronary artery disease, including reflecting plaque burden, predicting no-reflow events, predicting the prognosis of patients, reflecting LVR, and guiding the management of patients with MI. We hope that, in the near future, new studies will be conducted to better characterize and understand the relationship between sST2 and CAD.

## Author contributions

JZ and ZC wrote the manuscript. MM and YH conceived, instructed, reviewed, and revised the manuscript. All authors read and approved the final version of the manuscript.

## Funding

This work was supported by the Fellowship of China Postdoctoral Science Foundation (No. 2020M683325), the Postdoctoral Research Project, West China Hospital, Sichuan University (No. 2020HXBH048), and the Innovative Scientific Research Project of Medical Youth in Sichuan Province (No. Q20061).

## Conflict of interest

The authors declare that the research was conducted in the absence of any commercial or financial relationships that could be construed as a potential conflict of interest.

## Publisher's note

All claims expressed in this article are solely those of the authors and do not necessarily represent those of their affiliated organizations, or those of the publisher, the editors and the reviewers. Any product that may be evaluated in this article, or claim that may be made by its manufacturer, is not guaranteed or endorsed by the publisher.
